# Identification of QTLs for total flavonoid content and development of KASP markers in cultivated peanut (*Arachis hypogaea* L.)

**DOI:** 10.3389/fpls.2026.1945423

**Published:** 2026-10-30

**Authors:** Haoyu Zhang, Shunli Cui, Xinyu Li, Yingru Liu, Zhen Li, Lifeng Liu, Xiukun Li, Mingyu Hou

**Affiliations:** State Key Laboratory of North China Crop Improvement and Regulation, Key Laboratory for Crop Germplasm Resources of Hebei, North China Key Laboratory for Crop Germplasm Resources of Education Ministry, College of Agronomy, Hebei Agricultural University, Baoding, China

**Keywords:** GWAS, KASP marker, peanut, QTL mapping, RIL, Total flavonoid content

## Abstract

Flavonoids are important nutritional components, and total flavonoid content (TFC) is a critical quality trait in peanut (*Arachis hypogaea* L.) kernels. Identifying the genetic loci and candidate genes underlying TFC is essential for improving nutritional quality through molecular breeding. In this study, quantitative trait locus (QTL) mapping and genome-wide association study (GWAS) were integrated to explore the key loci of TFC in peanut. In a recombinant inbred line (RIL) population evaluated across four environments, TFC ranged from 46.96 to 131.15 mg rutin equivalents (RE)/100 g. A total of 11 QTLs associated with TFC were identified, among which *qflvA03.1* and *qflvA10.2* were major-effect loci, accounting for 16.95% and 11.64% of the phenotypic variation explained (PVE), respectively. In a natural population of 103 peanut accessions evaluated across three environments, TFC ranged from 63.49 to 129.27 mg RE/100 g. GWAS identified 92 significantly associated single-nucleotide polymorphisms (SNPs), explaining 9.15%-18.19% of the phenotypic variation. Four SNPs were co-localized with QTL intervals detected in the RIL population, distributed on chromosomes A06, A10, and B09, contributing 6.60%–8.9% to the PVE. Within 160 kb (*r^2^* = 0.2) regions surrounding these co-localized SNPs, 33 candidate genes potentially involved in flavonoid biosynthesis were identified, including genes encoding 4-coumarate-CoA ligase, isoflavone reductase, anthocyanin 5-aromatic acyltransferase, and flavonol synthase. A Kompetitive Allele-Specific PCR marker was developed at locus A03_124522117. The KASP marker was developed from the *qflvA03.1* QTL, and used to validate the association between genotype and flavonoid content. The genotypes carrying CC had significantly higher flavonoid content than those carrying TT. These findings provide insights into the genetic mechanisms underlying TFC in peanuts and establish a foundation for molecular breeding of high-quality, flavonoid-rich peanut varieties.

## 1 Introduction

Flavonoids are major bioactive components in peanut (*Arachis hypogaea* L.) kernels, characterized by a diphenylchromone backbone with a C6-C3-C6 carbon skeleton. Flavonoids exhibit strong free radical scavenging capacity and diverse biological functions, including antioxidant activity, immune modulation, and regulation of blood glucose, lipid metabolism, and blood pressure (Mondal et al., 2015). Accordingly, improving flavonoid content has become a key objective in functional peanut breeding.

TFC is a typical quantitative trait influenced by multiple loci and environmental factors (Liu et al., 2026). Classical genetic studies indicate that flavonoid content in peanut seeds is controlled by four pairs of major genes with additive effects, with heritability reaching 98.48% (Hou et al., 2020). In recent years, advances in molecular marker technologies and quantitative trait locus (QTL) mapping have facilitated the identification of loci associated with flavonoid content in various crops. Two important QTLs (*qTFC1–6* and *qTFC9-7*) related to flavonoid content have been identified in rice (Xia et al., 2023). Genome-wide association study (GWAS) of natural *Arabidopsis thaliana* accessions has linked variation in kaempferol 3-O-rhamnoside-7-O-rhamnoside and quercetin 3-O-rhamno-side-7-O-rhamnoside to *transparent testa 7* (*AT5G07990*) (Naake et al., 2024).In soybean, 29 isoflavone QTLs have been detected using an F_2_ population (Kim et al., 2023). Collectively, these studies indicate that flavonoid content is a typical quantitative trait governed by multiple genes and often exhibits significant genotype-by-environment interaction effects across plant species. In peanuts, a RIL population consisting of 121 lines derived from a cross between VG9514 and TAG 24 has been used for flavonoid content mapping. This leads to the identification of five major QTLs (Mondal et al., 2015). In addition, a major QTL *qIAA_A03_2* related to antioxidant activity of peanut is mapped with a PVE of 53.75% (Zhao et al., 2026).

The combination of QTL mapping and GWAS provides complementary advantages (Zhu et al., 2022; Zhang et al., 2026). For example, key loci and regulators of stem strength in soybean are identified through the integration of QTL mapping and GWAS (Liu et al., 2025). Through mutual validation between biparental populations and natural germplasm, this integrated strategy significantly improves the accuracy and stability of locus detection for complex traits such as pod size, seed weight, and drought tolerance, while narrowing candidate genomic regions and facilitating the identification of key candidate genes in peanut (Gangurde et al., 2020; Dang et al., 2024; Lu et al., 2025).

To further explore the genetic architecture of total flavonoid content (TFC) in peanut kernels, this study combined linkage mapping of RIL populations with differences in total flavonoid content (TFC) and genome-wide association analysis (GWAS) to identify candidate genes associated with TFC. By cross-validating RIL population QTL mapping and GWAS of natural germplasm, the accuracy of complex trait locus detection was improved, candidate intervals were refined, candidate genes were identified, and KASP markers were developed. These findings provide a basis for elucidating the molecular mechanisms underlying flavonoid biosynthesis in peanuts and support breeding programs aimed at developing high-flavonoid peanut varieties.

## 2 Materials and methods

### 2.1 Plant materials

The 103 peanut germplasm lines used for association analysis were obtained from the peanut breeding innovation team at Hebei Agricultural University. The materials were planted at the XuShui Experimental Base of Hebei Agricultural University (39.06° N, 115.61° E) in May 2021, May 2022, and May 2023 (designated as 2021XS, 2022XS, 2023XS, respectively) and harvested in September according to their respective maturity stages.

The parents of the RIL population were ‘Silihong’ (female parent) and ‘Jinonghei3’ (male parent), consisting of 227 families (Hao et al., 2022). The population was planted at the Qingyuan Experimental Base of Hebei Agricultural University (38.10° N, 113.40° E) in May 2018 and harvested in September of the same year (2018QY, E1). Additional trials were conducted at Xushui, Hebei (38.09° N, 115.46° E) (2021XS, E2) and Fuxin, Liaoning (42.01° N, 121.65° E) (2021FX, E3) in May 2021, with harvesting in September of the same year. The population was further evaluated at the Xushui Experimental Base of Hebei Agricultural University in May 2023, and plants were harvested in September of the same year (2023XS, E4).

Each germplasm line was planted in two rows with 10 holes in each row, one seed per hole. Each row was 1.5 m in length with an inter-row spacing of 42.5 cM. Three plants per line were harvested, naturally air-dried, and stored for later analysis.

### 2.2 TFC phenotyping

Aluminum chloride colorimetry was employed according to a previously reported protocol. A standard curve was constructed using rutin (purity 98%). The detailed parameters for aluminum chloride colorimetric assay have been supplemented in section 2.2, including extraction solvent, extraction temperature-time, solid-liquid ratio, AlCl_3_ concentration, reaction time and detection wavelength. Three replicates were included for each sample. TFC was expressed as mg rutin equivalents (RE) per 100 g (mg RE/100 g).

Statistical analyses were performed using SPSS Statistics 26 software, the linear model for two-way analysis of variance was $y_{ij}=\mu +G_{i}+E_{j}+\epsilon_{ij}$, where genotype was treated as a random effect, while environment was considered a fixed effect. Broad-sense heritability (*H^2^*^)^ was estimated using the formula $H^{2}=\sigma_{g}^{2}/\left(\sigma_{g}^{2}+\sigma_{e}^{2}/n\right)$. $\sigma_{g}^{2}$ indicates genotypic variance, $\sigma_{e}^{2}$ represents residual variance, and n is the number of test environments. Normality testing, analysis of variance (ANOVA), and frequency distribution mapping of TFC within the population were conducted.

### 2.3 GWAS

Genotyping of the RIL population was conducted using a SNP microarray (Affymetrix Axiom_Arachis2). Quality control of SNP markers was performed before GWAS. SNPs with minor-allele frequency (MAF)< 0.05, missing rate > 20%, and significant deviation from Hardy-Weinberg equilibrium (P< 1×10^-6^) were excluded. The chip contains a total of 47,837 SNP probes, evenly distributed across all 20 chromosomes of cultivated peanut, achieving good whole-genome coverage of both the A and B subgenomes. After quality control filtering, 35,416 high-quality SNPs were retained for subsequent genome-wide association analysis. The remaining high-quality SNPs were used for subsequent GWAS analysis. Based on the genotypic data obtained in the early stage of the laboratory and the results of population structure analysis (Li et al., 2022), GWAS was implemented using Tassel 5.0 software. A general linear model (GLM) was selected in the modeling process, and the generation of false positive correlation signals was effectively controlled by incorporating the population kinship matrix (K) and the population structure matrix (Q) as covariates. Using a multi-model comparison strategy, the influence of different covariate combinations on model fit was comprehensively evaluated, and the optimal analysis model was finally determined. The threshold of relevance significance was set to *P<* 1.0×10^-2^ (i.e., -log10(*P*)≥2.0). The R language platform was used to draw Q-Q plots and Manhattan plots.

### 2.4 QTL mapping

A RIL population derived from ‘Silihong’ and ‘Jinonghei3’ was previously constructed in our laboratory, and a high-density genetic linkage map was developed based on this population (Li et al., 2026). The map comprised 4,499 molecular markers distributed across 20 chromosomes, spanning a total length of 1712.32 cM and an average marker interval of 0.38 cM. Based on this genetic map, additive QTL mapping was performed using inclusive Composite Interval Mapping-Additive (ICIM-ADD) implemented in QTL IciMapping 4.2 software. Multi-environmental phenotypic data were used for mapping. A logarithm of odds (LOD) score threshold of ≥2.0 was set as the criterion for significance. The actual LOD threshold is 2.61. Genetic loci controlling TFC were systematically identified and analyzed.QTL analysis was performed using the ICIM-ADD module in QTL IciMapping V4.2, with a step size of 1.0 cM and a scanning window of 10.0 cM. The LOD threshold was determined by 1,000 permutation tests at α = 0.05.

### 2.5 Candidate gene screening and expression

The reference genome of Tifrunner was used for candidate gene identification (Bhat et al., 2023). Genomic regions extending 160 kb (*r^2^* = 0.2) upstream and downstream of each single-nucleotide polymorphism (SNP) were designated as the candidate interval, according to the reference linkage disequilibrium (LD) decay level. Candidate genes located within these intervals were screened.

Candidate gene expression profiles related to TFC were generated from transcriptome data (Deng et al., 2025). Transcriptome sequencing were used to analyze the regulatory mechanism of TFC during the drying process at five time points in parents. The transcriptome data of Silihong deposited in the NCBI SRA database (accession: PRJNA1212227). The transcriptome data of Jinonghei3 was unpublished.

### 2.6 Development and validation of KASP markers

The SNPs (Tifrunner.gnm2.ann1; Physical location: A03_124522117, Allelic mutation: T/C) within QTL intervals between Silihong and Jinonghei3 were identified based on resequencing data and used for KASP marker development. Primers were designed based on the differential SNP sequences identified. Primers were designed using Primer 5 software, The forward primer sequences (5’→3’) of KASP were GAAGGTGACCAAGTTCATGCTAGCTGCATTAGAAGCAGTTCAT and GAAGGTCGGAGTCAACGGATTAGCTGCATTAGAAGCAGTTCAC. The reverse primer sequence (5’→3’) was TCCCTCCAATCGTTTCACTACT. KASP assays were performed in 10 μL reaction volumes comprising 2.0 μL of genomic DNA (25–50 ng), 5 μL of 2×AQP master mix, 0.15 mmol L^-1^ of each forward primer, 0.4 mmol L^-1^ of the common reverse primer, and double-distilled water (Jason Gen Biotech Co. Ltd., Beijing, China). PCR amplification was conducted on an ABI QuantStudio6machine (ABI Life Technologies, CA, USA) under the following conditions: 60 °C for 30 s; 95 °C for 15 min; 95 °C for 20 s, 61 °C (-0.6 °C per cycle) for 40 s for 10 cycles; 95 °C for 20 s, 55 °C for 40 s for 40 cycles. Fluorescence signals were detected, and data analysis was performed using the built-in ABI QuantStudio6 software. The KASP markers were then evaluated in a peanut panel comprising 13 breeding lines and 13 cultivars and landraces from China.

## 3 Results

### 3.1 Phenotypic variation and environmental effects on TFC in peanut germplasm and RIL populations

The TFC of the parental lines, the RIL populations, and the GWAS population were evaluated and subjected to variance analysis under different environments (**Table 1**). In the RIL population, TFC showed substantial variation across environments. The widest range was observed in the 2018QY environment, ranging from 46.96 to 131.15 mg RE/100 g, while the highest coefficient of variation was detected in the 2021FX (E3) environment (16.64%).

**Table 1:** Phenotypic variation in flavonoid content in peanut.

Material	Environment	Range (RE mg/100 g)	Mean	SD	CV	Kurtosis	Parents (mg RE/100 g)	
							Silihong	Jinonghei3
RIL	E1(2018QY)	46.96-131.15	83.68	13.00	15.54	-0.019	76.11	92.54
	E2(2021XS)	60.06-124.96	95.23	12.64	13.27	-0.003	86.60	92.31
	E3(2021FX)	50.20-128.43	83.65	13.92	16.64	-0.105	77.29	89.43
	E4(2023XS)	65.70-121.55	95.20	12.76	13.40	-0.580	84.79	86.17
GWAS	2021XS	67.06-106.17	84.83	7.36	8.68	-0.139		
	2022XS	63.49-122.49	89.45	9.48	10.60	0.127		
	2023XS	63.98-129.27	95.09	12.29	12.92	-0.211		

Frequency distribution of total flavonoid content (TFC). RIL panels (blue) show the frequency distribution of TFC in recombinant inbred lines derived from the cross between Silihong and Jinonghei3 (S: Silihong; J: Jinonghei3).

In the GWAS population, TFC was evaluated in 2021, 2022, and 2023. The overall range was 63.49-129.27 mg RE/100 g, with average values of 84.83, 89.45, and 95.09 mg RE/100 g, respectively. TFC in 2023 was significantly higher than that in the other two environments (**Figure 1**).

**Figure 1:**
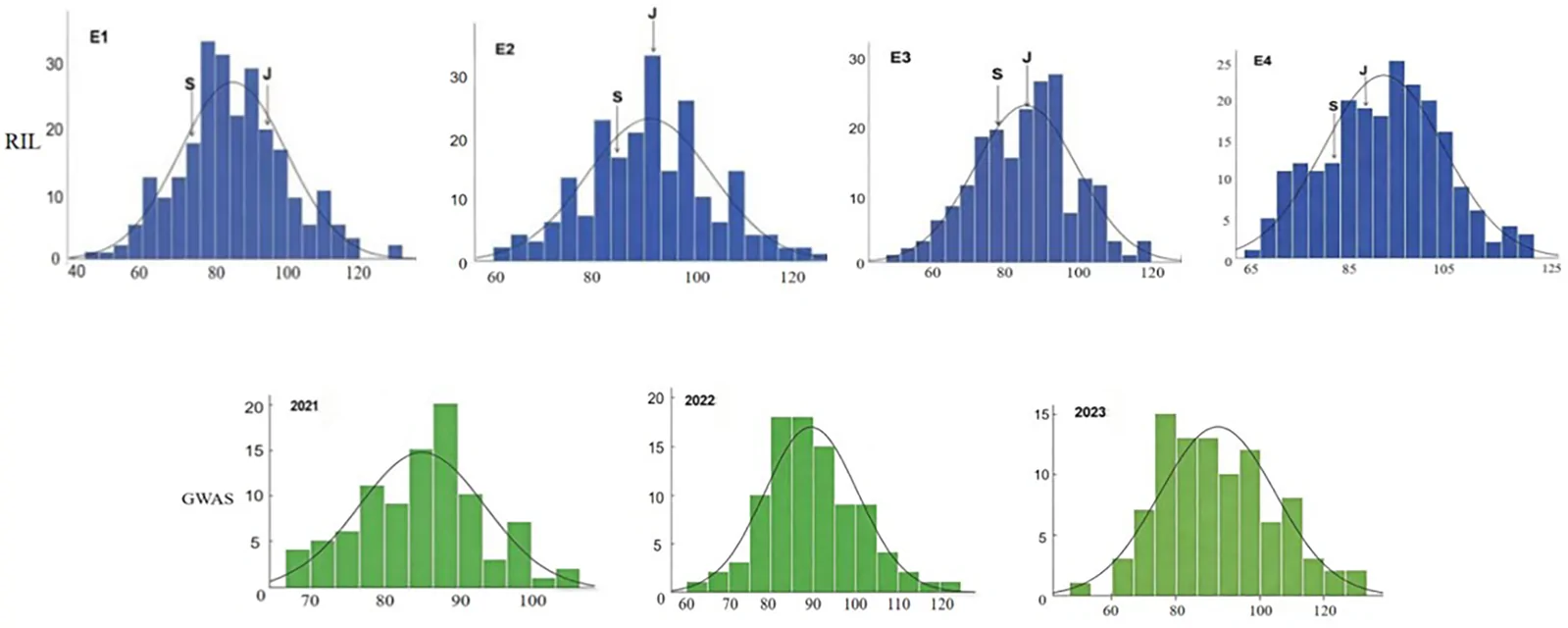
Frequency distribution of total flavonoid content. E1, E2, E3, and E4 represent four environments 2018QY, 2021XS, 2021FX, and 2023XS, respectively. GWAS panels (green) show the frequency distribution of TFC in the natural population. The years 2021, 2022, and 2023 correspond to environments 2021XS, 2022XS, and 2023XS, respectively. S: Silihong; J: Jinonghei3.

The broad-sense heritability of the RIL population was 60.36%, higher than that of the GWAS population 58.64%, and both genotype and environment had significant effects on the flavonoid content of the RIL and GWAS populations (**Supplementary Table S1**).

### 3.2 Genome-wide genetic dissection of TFC through QTL mapping and SNP-based association analysis

#### 3.2.1 Identification of stable and major-effect QTLs for TFC across multiple environments in the RIL population

QTLs associated with flavonoid content were analyzed in the RIL population across four environments. QTLs with LOD values greater than 2 were considered significant. A total of 11 QTLs associated with flavonoid content were identified, primarily distributed across seven chromosomes (A03, A06, A08, A10, B01, B08, and B09) (**Supplementary Table S3**; **Figure 2**). Notably, two major QTLs were detected in the E4 environment. The QTL *qflvA03.1*, located on chromosome A03, had a LOD value of 11.51, with a PVE of 16.95%. Its additive effect was positive, indicating that the favorable allele originated from the male parent. The second major QTL, *qflvA10.2*, located on chromosome A10, had a LOD value of 8.24 and accounted for 11.64% of the PVE. Its negative additive effect indicated that the favorable allele was derived from the female parent. The two major QTLs provide valuable loci for marker-assisted selection and genetic improvement of flavonoid content in peanut.

**Figure 2:**
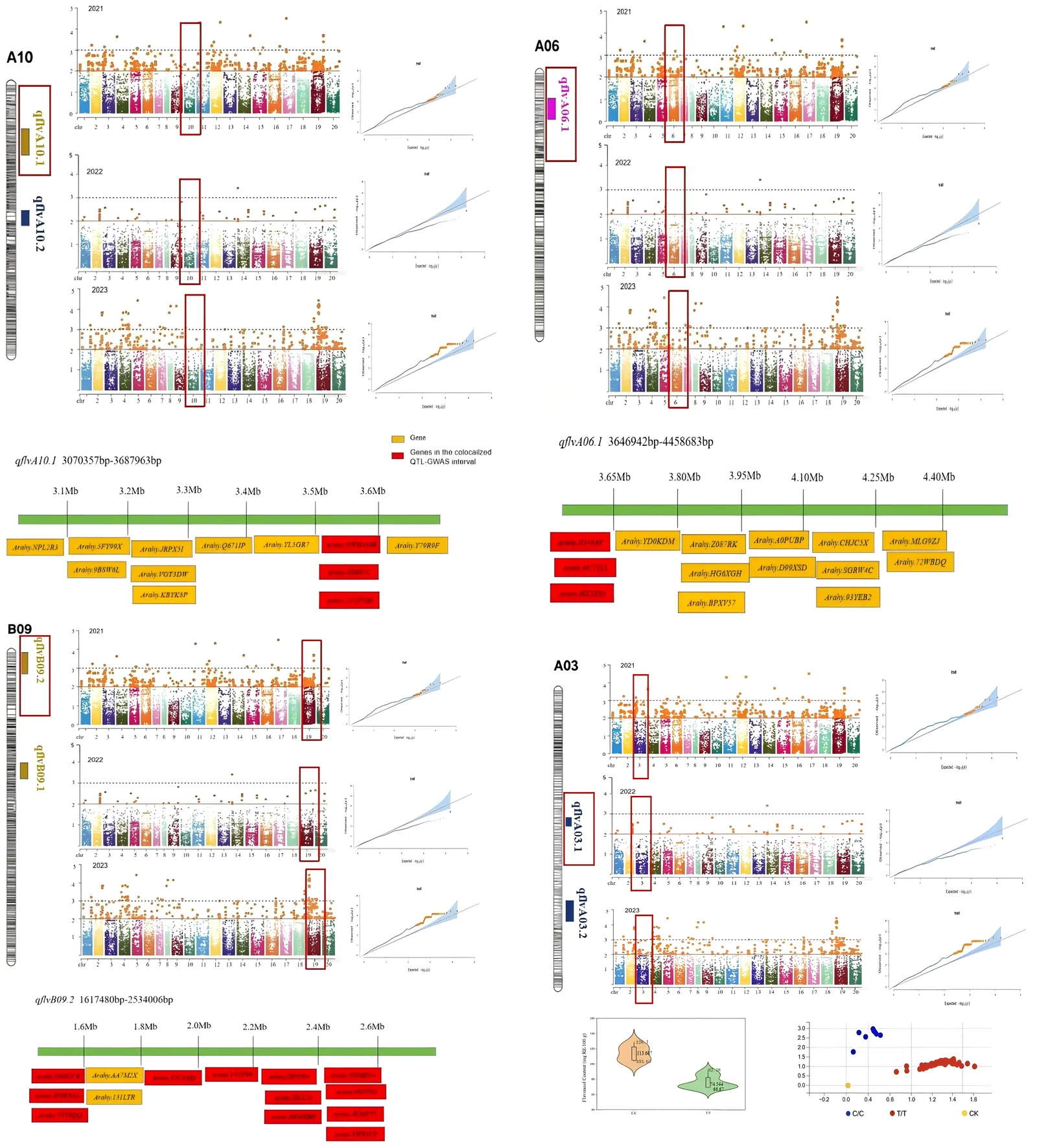
Chromosomal distribution of QTLs and candidate genes associated with total flavonoid content in peanut. QTL distribution map: QTLs associated with total flavonoid content detected in four environments using the RIL population derived from the cross between Silihong and Jinonghei3. E1, E2, E3, and E4 correspond to 2018QY, 2021XS, 2021FX, and 2023XS, respectively. Candidate gene distribution map: Distribution of candidate genes within co-localized intervals (*qflvA06.1*, *qflvA10.1*, *qflvB09.2*) identified from the RIL population (with Silihong and Jinonghei3 as parents) and GWAS across the corresponding chromosomal interval. Genes marked with red boxes indicate candidate genes located at loci mapped in the RIL population for the SNPs identified by GWAS. Genes marked with yellow boxes indicate candidate genes located within 160 kb upstream and downstream of the co-localized intervals between the RIL population and GWAS, including flavonoid-related candidate genes within *qflvA03.1*, which exhibited the highest PVE (16.95%) among QTLs identified in the RIL population.

#### 3.2.2 GWAS revealing significant SNPs and genomic regions associated with TFC variation

The optimal experimental model was selected based on the Q-Q plot, and SNPs with *p*-values below the significance threshold were considered associated. A total of 323 SNPs were detected across the three environments, among which 92 were significantly associated with TFC. These significant SNPs were distributed across 17 chromosomes, including A02, A04, A05, A07, and B09, with PVEs ranging from 9.15% to 18.19%. Among these, 30 SNPs were located on chromosome B09, with PVEs ranging from 10.44% to 17.27%. Notably, four SNPs were clustered between 17,225,859 bp and 17,997,756 bp, with PVEs between 10.88% and 15.98%. (**Supplementary Table S2**; **Figure 2**).

Among the detected loci, seven SNPs were consistently identified in at least two environments (**Supplementary Table S3**). These were distributed across four chromosomes (A04, A05, A09, and B09), with PVEs ranging from 6.45% to 13.12%. The SNP AX-147234336 on chromosome A09 exhibited the highest PVE (13.12%) in 2022. Additionally, three closely linked SNPs (positions 27,446,871-28,017,021 bp) on chromosome B09 were detected in 2021 and 2023, with PVEs ranging from 7.68% to 9.52%.

### 3.3 Co-localization of QTLs between the natural and RIL population

In this study, four SNPs identified in the natural population coincided with QTL loci detected in the RIL population (**Supplementary Table S3**). These co-localized SNPs were distributed on chromosomes A06, A10, and B09, contributing 6.60%-8.95% of the PVE. Within the *qflvA06.1* QTL interval, two SNPs, AX-176793905 and AX-176801436, were identified on chromosome A06. Both SNPs were detected in 2021, accounting for 6.60%-6.71% of the PVE.

### 3.4 Candidate gene mining within major and co-localized SNPs

Based on the *Arachis hypogaea* genome (Tifrunner v2.0), 33 candidate genes related to flavonoid metabolism were identified within the 160 kb (*r^2^* = 0.2) regions surrounding the significant and co-localized SNPs (**Supplementary Table S4**; **Figure 3**). Among them, structural genes involved in flavonoid biosynthesis included 4- coumaric acid coenzyme A, isoflavone reductase, anthocyanin 5-aromatic acyltransferase, and ketol synthase. Genes involved in post-modification of flavonoids included various transferases and glycosyltransferases. Transcription factors potentially regulating flavonoid biosynthesis included members of the MYB, MYC, BHLH, and WD40 families. The expression levels of 24 candidate genes, including *Arahy.42MKBI*, showed marked differences between the two parents across different drying stages (**Figure 3**).

**Figure 3:**
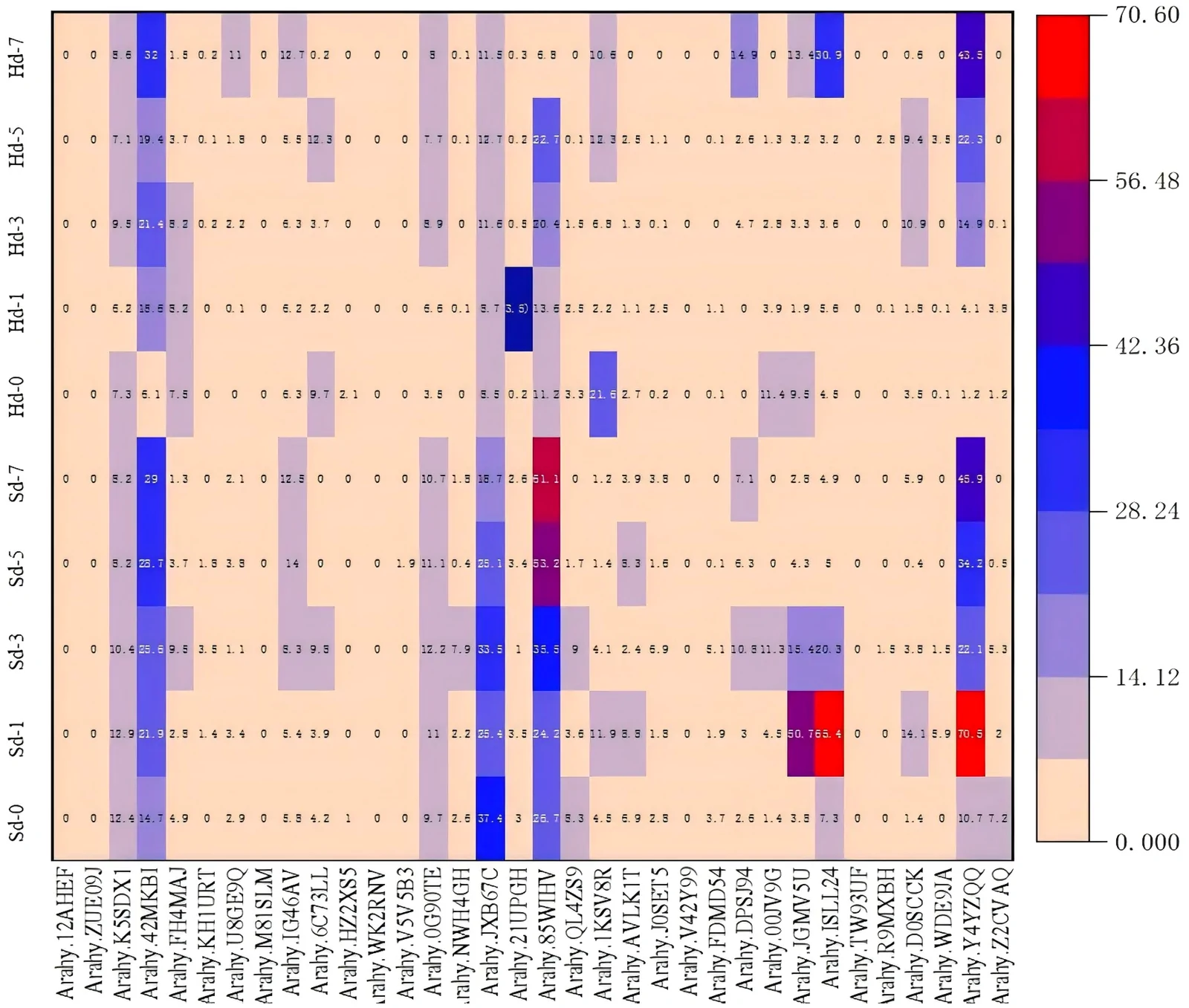
Expression profiling of candidate genes using transcriptome data. Sd-0, Sd-1, Sd-3, Sd-5, and Sd-7 represent samples of the parental line Silihong dried for 0, 1, 3, 5, and 7 days, respectively; Hd-0, Hd-1, Hd-3, Hd-5, and Hd-7 represent samples of the parental line Jinonghei dried for 0, 1, 3, 5, and 7 days, respectively.

### 3.5 Validation of KASP marker associated with *qflvA03.1* and flavonoid content variation

A KASP marker developed from the Arahy.A03 (LOD = 11.51, PVE = 16.95%, physical interval: 124374666-125285413) locus within the *qflvA03.1* QTL region was used to validate the association between genotype and flavonoid content. This interval spanned 124.37-125.29 Mb, and the marker interval was 511–512. This marker was selected for KASP assay because it exhibits high polymorphism among peanut germplasm and is located within *Arahy.K5SDX1*. The results showed that accessions carrying the CC genotype had significantly higher flavonoid content than those carrying the TT genotype (F = 159.17, P< 0.01) (**Figure 2**; **Supplementary Tables S3**, **S5**). The statistical results of genotypes and flavonoid content for each material are as follows: TFC in CC genotypic materials ranged from 103.67 mg RE/100 g to 128.70 mg RE/100 g, with an average of 113.607 mg RE/100 g; TFC in TT genotypic materials ranged from 66.67 mg RE/100 g to 92.38 mg RE/100 g, with an average of 74.544 mg RE/100 g (**Table 2**) These results indicate that the developed KASP markers can be effectively used for screening and identifying flavonoid content in peanuts.

**Table 2:** Statistical results of peanut genotypes and flavonoid content.

Mater number	Genotype	Flavonoid content(mg RE/100g)
RIL-231	C/C	117.50
Yi xian yi wo hou	C/C	104.67
Zhuo xian yi ba jiu	C/C	105.40
Fu ning da li	C/C	106.98
Kai nong 58	C/C	103.67
M3	C/C	120.814
M51	C/C	123.84
M67	C/C	128.70
C76-16	C/C	110.89
Fu ning da li	T/T	92.38
Huang ye hong hua	T/T	82.37
He li ba tuo	T/T	81.53
Nan hun san li	T/T	68.96
Feng run da hua sheng	T/T	87.66
Quan hua 361	T/T	72.98
RIL-1	T/T	69.38
RIL-40	T/T	66.94
RIL-42	T/T	74.50
RIL-52	T/T	70.63
RIL-65	T/T	72.21
RIL-68	T/T	70.70
RIL-81	T/T	71.25
RIL-98	T/T	72.17
RIL-132	T/T	69.56
RIL-133	T/T	71.82
RIL-161	T/T	88.89
RIL-186	T/T	86.02
RIL-188	T/T	66.67
RIL-197	T/T	70.65
RIL-202	T/T	68.93
RIL-205	T/T	72.65
RIL-208	T/T	77.21
RIL-209	T/T	66.89
RIL-241	T/T	70.65

## 4 Discussion

Flavonoids are important secondary metabolites in plants, and their synthesis and accumulation are influenced by environmental factors, including illumination, temperature, humidity. For example, flavonoid content in maize leaves treated with enhanced UV-B radiation was significantly higher than in plants under natural light (Righini et al., 2019). Similarly, flavonoid levels in cowpeas showed significant correlations with temperatures and precipitation (Mweta et al., 2025). In jujube fruits, TFC was positively correlated with temperature (Duan et al., 2023). In the GWAS population of this study, the average flavonoid content in peanut seeds in 2023 was 95.09 mg RE/100 g. That year, Baoding experienced higher-than-average sunshine duration, greater precipitation, and record-high summer temperatures (data source: National Bureau of Statistics). These environmental conditions likely stimulated peanut plants to increase flavonoid synthesis as a physiological response to environmental stress. This finding is consistent with previous studies in other crops, reinforcing that climatic conditions play an important role in regulating the accumulation of secondary metabolites in plants. To accurately dissect this genetic architecture and separate locus-specific effects, multi-environment trials are necessary.

Several QTLs and candidate genes associated with flavonoid-related traits have been previously identified in peanut (**Supplementary Table S6**). The major QTL *qflvA03.1* was located between markers 511–512 on chromosome A03, and another locus *qflvA03.2* was located between markers 533–534 downstream of *qflvA03.1*, with a small contribution rate of 2.75%. It is noteworthy that *qflvA03.1* with 16% phenotypic variance explained was identified despite small phenotypic differences between the two parents. This phenomenon likely arises from compensatory genetic effects in parental backgrounds, where allele effects are masked by epistasis or other counteracting loci. Recombination in the segregating population uncovers the locus effect. Potential PVE overestimation from the Beavis effect and merged signals of linked QTLs should not be ignored; multi-environment trials are needed to confirm its reliability. However, these two loci were detected only in the E4 environment. Notably, a major QTL, *qFlv2010-a03*, was mapped to chromosome A03, with a LOD score of 3.15 and 25.2% PVE (Mondal et al., 2015). However, this locus was not detected across multiple environments. Since that study was based on an SSR marker-based linkage map, it is not possible to confirm whether this locus is identical to the one identified in the present study, although its strong genetic effect on flavonoid content in peanut seeds has been demonstrated. Furthermore, it is noteworthy that the locus identified in this study shows a similar environmental specificity to *qFlv2010-a03*, as it was also detected only in the E4 environment. The KASP marker developed for this locus enables the discrimination of germplasm accessions with different levels of TFC. This environmental specificity suggests that environmental factors may play a crucial role in regulating the expression of this locus, and the underlying genes are likely to exhibit environment-dependent expression patterns that influence flavonoid biosynthesis. Considering that such environment-dependent expression is a common feature of quantitative traits in plants, environmental factors should be incorporated into future studies on the functional characterization and regulatory mechanisms of this locus. Mondal et al. (2015) identified five major-effect QTLs controlling flavonoid content in peanut seeds, among which qFlv2010-a03 on chromosome A03 could explain 25.2% of the phenotypic variation; however, this locus was detected only under a single environment. Both A03 loci exhibited significant environmental specificity and were only identified in individual trial environments. Mondal et al. (2015) did not detect any major-effect flavonoid-related QTLs on chromosome A10, whereas in this study, a new major-effect QTL *qflvA10.2* (PVE = 11.64%, 109.6-110.2 Mb) was identified on chromosome A10. This genomic interval has been reported to contain seed coat pigment-related genes, further indicating that chromosome A10 is involved in regulating the metabolic flux between flavonols and anthocyanins in peanut seeds.

The major QTL *qflvA10.2* identified in this study provides a key genetic resource for dissecting the molecular mechanism underlying flavonoid biosynthesis in peanut seeds. A seed coat color gene was identified within the 108-112.7 Mb interval of chromosome 10 (A10) (Zhao et al., 2021), and the seed coat pigmentation locus *AhTc1* was mapped near marker J3K16K on the same chromosome (Lu et al., 2024). In alignment with these findings, our identified *qflvA10.2* is localized within the 109.6-110.2 Mb interval of chromosome A10, while a minor QTL *qflvA10.1* (detected exclusively in the E1 environment) was mapped between markers 1491 and 1492. Collectively, Collectively, these results suggest that chromosome A10 may harbor genes involved in flavonoid metabolism. Notably, *qflvA10.1* and *qflvA10.2* exhibit additive effects derived from the two parents (Jinonghei3 with a black seed coat and Silihong with a red seed coat), which supports the hypothesis that the A10 chromosome region is associated with the metabolic shift from quercetin to anthocyanin-two core flavonoid compounds sharing common biosynthetic pathways. This study not only enriches the genetic regulatory network of flavonoid biosynthesis in peanuts but also lays a solid foundation for exploring the genetic relationship between quercetin and anthocyanin. Further fine mapping and gene cloning of these QTLs will help identify key regulatory genes and deepen our understanding of the molecular mechanisms underlying flavonoid metabolism in peanuts.

Three SNPs (27446871–28017021 bp) on peanut chromosome B09, accounting for 7.68%-9.52% of the PVE, were clustered within a specific interval. Notably, the SNP locus AX-177642604, which was co-localized between the RIL and natural populations on chromosome B09, highlights the presence of a key regulatory region controlling flavonoid content. As plant secondary metabolites, flavonoids are closely linked to biotic stress, and chromosome B09 has previously been associated with multiple stress resistance loci in peanut, including the leaf spot resistance QTL *9AUDPC.B09_37.0* at 37.0 cm (Gomis et al., 2025), the cold resistance locus *qRGRB09* (155637831–155854093 bp) during germination (Zhang X. et al., 2023), and the tomato leaf blight resistance QTL *qTSW_T10_B09_2* (59.2-64.4 cM, 40.7% PVE) (Agarwal et al., 2018). These findings confirm that there may be key genes on chromosome B09 that regulate flavonoid metabolism and stress tolerance. Further functional analysis of these genes is crucial for elucidating the molecular mechanisms of flavonoid biosynthesis in peanuts.

Flavonoid accumulation is a complex quantitative trait controlled by major and minor genes (Lepiniec et al., 2006), and is regulated by structural genes (*PAL*, *CHS*, *C4H*, *CHI*) and transcription factors. GWAS and QTL mapping in this study identified multiple loci and candidate genes (**Supplementary Table S4**), including key components of the flavonoid biosynthetic pathway and its regulatory networks. Structural genes included *Arahy.1KSV8R* (encoding 4-coumarate-CoA ligase, 4CL), which catalyzes the conversion of coumaric acid to coumaroyl-CoA (a precursor of flavonoid) and regulates carbon flux between flavonoid and lignin biosynthesis pathways, and *Arahy.12AHEF* (encoding flavonol synthase, FLS) (Ma et al., 2025), which mediates the conversion of dihydroflavonol-to-flavonol and exhibits evolutionarily conserved function. Genes encoding flavonoid post-modification enzymes, such as anthocyanin 5-aromatic acyltransferase, methyltransferase, and glycosyltransferase, were also identified; these enzymes contribute to the diversification of flavonoid structure, stability, and bioactivity (Hou et al., 2020; Li et al., 2025). The cytochrome P450 family gene *Arahy.QL4ZS9* was mapped to chromosome B02. High expression of the cytochrome P450 family gene *ah8f7pe4* could enhance F3’H enzyme activity, thereby promoting anthocyanin accumulation, whereas low expression of *ahk8h9r8* may reduce its contribution to anthocyanin synthesis (Zhang K. et al., 2023). Transcription factors (MYB, MYC, bHLH, and WD40) were also identified, which regulate flavonoid biosynthesis by binding to cis-acting elements in the promoters of structural genes (Hichri et al., 2011). MYB/bHLH-mediated gene expression has been reported to be associated with hormone-related flavonoid variation in peanuts (Gong et al., 2024).

## Data Availability

The datasets presented in this study can be found in online repositories. The names of the repository/repositories and accession number(s) can be found in the article/**Supplementary Material**.
