# Supplementary material for: Identification of QTLs for total flavonoid content and development of KASP markers in cultivated peanut (Arachis hypogaea L.)
Source: Front Plant Sci. 2026 Oct 30;17:1945423. doi: 10.3389/fpls.2026.1945423 (PMC13759822; doi:10.3389/fpls.2026.1945423)
Supplement: Supplementary file 1 [file DataSheet1.docx]

Table S1. Analysis of variance and heritability of flavonoid content phenotypes in natural peanut populations and RIL populations

| Material | Source of variation | df | SS | MS | F-Value | P-Value | H² |
| --- | --- | --- | --- | --- | --- | --- | --- |
| GWAS | Environment | 2 | 4023.140 | 2011.57 | 29.339 | <0.005 |  |
|  | Genotype | 102 | 17067.424 | 167.328 | 2.441 | 0.049 | 58.64 |
|  | Environment×Genotype | 181 | 18294.750 | 101.076 | 1.474 | 0.589 |  |
|  | Error | 92 | 10346.659 | 112.464 |  |  |  |
|  | Environment | 3 | 15624.615 | 5208.205 | 55.009 | ＜0.01 |  |
| RIL | Genotype | 236 | 52341.024 | 221.784 | 2.342 | 0.034 | 60.36 |
|  | Environment×Genotype | 153 | 15691.68 | 102.56 | 1.083 | 0.543 |  |
|  | Error | 135 | 12781.8 | 94.68 |  |  |  |

Note: The three GWAS environments were 2021XS, 2022XS, and 2023XS. The four RIL environments (E1–E4) corresponded to 2018QY, 2021XS, 2021FX, and 2023XS, respectively.

Table S2. SNPs significantly associated with flavonoid content in peanuts.

| Marker | Chromosome | Position | -log10(p) | R^2^（%） |
| --- | --- | --- | --- | --- |
| AX-176809111 | A01 | 92242189 | 3.20 | 13.57% |
| AX-147212613 | A02 | 2117440 | 3.22 | 9.77% |
| AX-147215223 | A02 | 92566325 | 3.83 | 15.97% |
| AX-176813902 | A02 | 86343809 | 3.83 | 15.96% |
| AX-176793136 | A02 | 86865465 | 3.71 | 15.51% |
| AX-176792250 | A03 | 22549013 | 3.15 | 9.37% |
| AX-176813202 | A04 | 1311622 | 3.63 | 10.93% |
| AX-176815008 | A04 | 68595247 | 3.86 | 13.41% |
| AX-176820331 | A04 | 37322175 | 3.82 | 13.42% |
| AX-176820672 | A04 | 92473425 | 3.79 | 13.14% |
| AX-176815360 | A04 | 22606633 | 3.47 | 14.65% |
| AX-177644634 | A04 | 88711456 | 3.24 | 13.73% |
| AX-176822521 | A04 | 95308697 | 3.22 | 13.63% |
| AX-176822195 | A04 | 97476552 | 3.22 | 13.63% |
| AX-176819415 | A04 | 95000363 | 3.22 | 13.63% |
| AX-176807533 | A04 | 93677054 | 3.22 | 13.63% |
| AX-176821601 | A04 | 65776229 | 3.22 | 13.62% |
| AX-176820307 | A04 | 70435647 | 3.22 | 13.62% |
| AX-176819174 | A04 | 34079632 | 3.22 | 13.62% |
| AX-176814683 | A04 | 87318281 | 3.22 | 13.62% |
| AX-176811999 | A04 | 96894328 | 3.22 | 13.62% |
| AX-176804831 | A04 | 34901336 | 3.22 | 13.62% |
| AX-176822778 | A04 | 62654130 | 3.20 | 13.54% |
| AX-176821264 | A04 | 95411898 | 3.17 | 13.62% |
| AX-176823207 | A04 | 84458505 | 3.05 | 10.41% |
| AX-147220383 | A04 | 96463503 | 3.05 | 10.33% |
| AX-176804179 | A04 | 88098426 | 3.03 | 12.90% |
| AX-176798573 | A04 | 78269593 | 3.03 | 12.90% |
| AX-176797235 | A04 | 36967796 | 3.03 | 12.90% |
| AX-176797560 | A04 | 6029070 | 3.02 | 12.86% |
| AX-176821524 | A04 | 102881142 | 3.02 | 12.86% |
| AX-176810839 | A05 | 69761830 | 3.08 | 9.38% |
| AX-176803856 | A05 | 79661825 | 3.08 | 9.38% |
| AX-176807342 | A05 | 46111930 | 4.42 | 18.19% |
| AX-147222599 | A05 | 69604244 | 3.20 | 10.96% |
| AX-176815040 | A06 | 72848330 | 3.18 | 9.68% |
| AX-177641063 | A07 | 14518932 | 3.13 | 10.59% |
| AX-147228413 | A07 | 53279095 | 3.06 | 13.02% |
| AX-177637963 | A07 | 76276161 | 3.03 | 12.90% |
| AX-177638491 | A08 | 25730809 | 4.15 | 17.17% |
| AX-177639886 | A08 | 12429536 | 3.83 | 15.97% |
| AX-177637425 | A09 | 16398948 | 4.15 | 17.17% |
| AX-176805719 | A09 | 118105918 | 3.10 | 10.49% |
| AX-176818825 | A09 | 18229713 | 3.00 | 10.56% |
| AX-176805662 | B01 | 29980953 | 4.30 | 13.22% |
| AX-147241989 | B02 | 93585680 | 4.32 | 13.27% |
| AX-176816597 | B02 | 23191567 | 3.37 | 10.42% |
| AX-147240077 | B02 | 1637548 | 3.22 | 9.77% |
| AX-176810340 | B02 | 2318148 | 3.18 | 9.60% |
| AX-147248574 | B04 | 127790198 | 3.68 | 13.62% |
| AX-176808262 | B04 | 4991298 | 3.41 | 15.63% |
| AX-176799653 | B05 | 111979532 | 3.11 | 9.50% |
| AX-176807662 | B05 | 10389151 | 3.07 | 9.24% |
| AX-176818757 | B06 | 120849648 | 3.17 | 9.62% |
| AX-176810778 | B06 | 107037899 | 3.14 | 13.37% |
| AX-176808206 | B07 | 30842126 | 4.50 | 14.51% |
| AX-176799158 | B07 | 27832734 | 3.02 | 9.15% |
| AX-177644365 | B08 | 121639693 | 3.54 | 12.12% |
| AX-177642341 | B08 | 123805023 | 3.54 | 12.12% |
| AX-177641806 | B08 | 123812069 | 3.38 | 11.63% |
| AX-177644351 | B08 | 103242906 | 3.06 | 13.02% |
| AX-177640589 | B08 | 96294160 | 3.06 | 13.02% |
| AX-177637342 | B09 | 132225231 | 3.71 | 11.32% |
| AX-177639066 | B09 | 131731036 | 3.65 | 11.02% |
| AX-177644205 | B09 | 133615583 | 3.39 | 12.87% |
| AX-177641592 | B09 | 17658500 | 4.43 | 15.48% |
| AX-177642417 | B09 | 24336963 | 4.25 | 15.05% |
| AX-147260782 | B09 | 25301106 | 4.18 | 17.27% |
| AX-177644185 | B09 | 10147019 | 4.15 | 17.17% |
| AX-177643630 | B09 | 22481492 | 4.15 | 17.17% |
| AX-177643163 | B09 | 25204171 | 4.15 | 17.17% |
| AX-177642991 | B09 | 23836642 | 4.15 | 17.17% |
| AX-177642595 | B09 | 25547926 | 4.15 | 17.17% |
| AX-177642458 | B09 | 22321426 | 4.15 | 17.17% |
| AX-177642154 | B09 | 22830206 | 4.15 | 17.17% |
| AX-177641669 | B09 | 21840344 | 4.10 | 17.18% |
| AX-177643628 | B09 | 18588406 | 3.83 | 15.98% |
| AX-177643604 | B09 | 18386171 | 3.83 | 15.98% |
| AX-177643193 | B09 | 17225859 | 3.83 | 15.98% |
| AX-177643031 | B09 | 19856290 | 3.83 | 15.98% |
| AX-177642227 | B09 | 18595871 | 3.83 | 15.98% |
| AX-177640686 | B09 | 17997756 | 3.83 | 15.98% |
| AX-147260645 | B09 | 19422512 | 3.83 | 15.98% |
| AX-177643905 | B09 | 23593690 | 3.77 | 15.86% |
| AX-147260589 | B09 | 17571193 | 3.15 | 10.88% |
| AX-177644088 | B09 | 86034009 | 3.10 | 10.49% |
| AX-177643470 | B09 | 99295459 | 3.10 | 10.49% |
| AX-177642591 | B09 | 54714174 | 3.10 | 10.49% |
| AX-177641411 | B09 | 107225815 | 3.10 | 10.49% |
| AX-177643736 | B09 | 109630028 | 3.05 | 10.50% |
| AX-147260646 | B09 | 19425414 | 3.04 | 10.49% |
| AX-177643501 | B09 | 10735224 | 3.04 | 10.44% |

| Table S3. Quantitative trait loci (QTL) and SNPs associated with TFC in peanut | | | | | | | | |
| --- | --- | --- | --- | --- | --- | --- | --- | --- |
|  | QTL | Environment | Chromosome | Position | PVE (%) | Marker interval | LOD | Additive |
|  | *qflvA08.1* | e1 | A08 | 37924617-38534599 | 3.58 | 1257-1258 | 2.31 | -31.16 |
|  | ***qflvA10.1*** | **e1** | **A10** | **3070357-3687963** | **3.49** | **1491-1492** | **2.36** | **30.53** |
|  | *qflvB09.1* | e1 | B09 | 156293993-157004269 | 5.03 | 4197-4198 | 3.19 | 36.68 |
|  | ***qflvB09.2*** | **e1** | **B09** | **1617480-2534006** | **3.86** | **3910-3911** | **2.18** | **-32.07** |
|  | ***qflvA06.1*** | **E2** | **A06** | **3646942-4458683** | **5.03** | **169-170** | **2.09** | **27.14** |
| RIL | *qflvB01.1* | E2 | B01 | 148170483-148882192 | 3.43 | 2016-2017 | 2.12 | -33.41 |
|  | *qflvB08.1* | E2 | B08 | 133083267-133817208 | 5.98 | 3906-3907 | 3.08 | -43.63 |
|  | *qflvA02.1* | E3 | A02 | 97765494-98458584 | 5.85 | 341-342 | 2.24 | 32.88 |
|  | ***qflvA03.1*** | **E4** | **A03** | **124374666-125285413** | **16.95** | **511-512** | **11.51** | **56.02** |
|  | *qflvA03.2* | E4 | A03 | 141190822-141841604 | 2.75 | 533-534 | 2.07 | 22.55 |
|  | *qflvA10.2* | E4 | A10 | 109583430-110171765 | 11.64 | 1711-1712 | 8.24 | -46.48 |
|  | AX-176812660 | 2022XS | A04 | 111718457 | 6.96 |  |  |  |
|  |  | 2023XS |  |  | 6.45 |  |  |  |
|  | AX-176808010 | 2021XS | A05 | 11985935 | 6.83 |  |  |  |
|  |  | 2022XS |  |  | 8.89 |  |  |  |
|  | AX-147222599 | 2022XS | A05 | 69604244 | 7.48 |  |  |  |
|  |  | 2023XS |  |  | 10.96 |  |  |  |
| GWAS | AX-147234336 | 2022XS | A09 | 113715433 | 13.12 |  |  |  |
|  |  | 2023XS |  |  | 9.45 |  |  |  |
|  | AX-177642583 | 2021XS | B09 | 27948368 | 7.68 |  |  |  |
|  |  | 2023XS |  |  | 9.52 |  |  |  |
|  | AX-177641390 | 2021XS | B09 | 27446871 | 7.68 |  |  |  |
|  |  | 2023XS |  |  | 9.52 |  |  |  |
|  | AX-177641115 | 2021XS | B09 | 28017021 | 7.68 |  |  |  |
|  |  | 2023XS |  |  | 9.52 |  |  |  |
|  | **AX-176793905** | **2021XS** | **A06** | **3652948** | **6.71** |  |  |  |
|  | **AX-176801436** |  | **A06** | **3696787** | **6.60** |  |  |  |
|  | **AX-147235101** | **2021XS** | **A10** | **3567092** | **8.95** |  |  |  |
|  | **AX-177642604** | **2023XS** | **B09** | **1781087** | **7.32** |  |  |  |

Note: QTLs in bold indicate co-localized loci identified in both GWAS and RIL analyses.

Table S4. Candidate genes related to flavonoid biosynthesis at the co-localized loci in peanut

| SNP | Chr. | Candidate gene | Function annotation |
| --- | --- | --- | --- |
| AX-176803831 | A01 | *Arahy.12AHEF* | Flavonol synthase/Flavanone 3-hydroxylase-like |
| AX-147212613 | A02 | *Arahy.ZUE09J* | UDP-Glycosyltransferase superfamily protein |
| AX-176813902 | A02 | *Arahy.42MKBI* | MYB transcription factor |
| AX-176818825 | A09 | *Arahy.FH4MAJ* | Gibberellin 20 oxidase 1-like |
| AX-176821264 | A04 | *Arahy.KH1URT* | Alpha 1,4-glycosyltransferase family protein |
| AX-176821524 | A04 | *Arahy.U8GE9Q* | Ethylene-responsive transcription factor 13-like |
| AX-176822778 | A04 | *Arahy.M81SLM* | Zinc finger MYM-type protein |
| AX-176793905 | A06 | *Arahy.IG46AV* | Pentatricopeptide repeat (PPR) superfamily protein |
| AX-176801436 | A06 | *Arahy.6C73LL* | Zinc ion binding |
|  |  | *Arahy.HZ2XS5* | Zinc finger CCCH domain protein |
| AX-177637963 | A07 | *Arahy.WK2RNV* | Cytochrome P450 superfamily protein |
| AX-177641063 | A07 | *Arahy.V5V5B3* | bHLH51-like |
|  |  | *Arahy.0G90TE* | Pentatricopeptide repeat (PPR) superfamily protein |
| AX-147235101 | A10 | *Arahy.NWH4GH* | Threonine kinase 2 |
|  |  | *Arahy.JXB67C* | G-type lectin S-receptor-like Serine/Threonine-kinase |
|  |  | *Arahy.21UPGH* | Receptor kinase 3 |
| AX-147240077 | B02 | *Arahy.85WIHV* | MYB114 isoform X2 |
|  |  | *Arahy.QL4ZS9* | Cytochrome P450 superfamily protein |
| AX-176810340 | B02 | *Arahy.1KSV8R* | 4-coumarate:CoA ligase 2 |
|  |  | *Arahy.AVLK1T* | Isoflavone reductase homolog |
|  |  | *Arahy.J0SET5* | UDP-Glycosyltransferase superfamily protein |
| AX-177639066 | B09 | *Arahy.V42Y99* | Anthocyanin 5-aromatic acyltransferase-like |
|  |  | *Arahy.FDMD54* | Disease resistance protein (TIR-NBS-LRR class) |
| AX-177642154 | B09 | *Arahy.DPSJ94* | MYB transcription factor |
| AX-177642595 | B09 | *Arahy.00JV9G* | MYC-type, basic helix-loop-helix (bHLH) domain |
| AX-177642991 | B09 | *Arahy.JGMV5U* | MYB transcription factor |
|  |  | *Arahy.ISLL24* | alpha-galactosidase |
| AX-177643163 | B09 | *Arahy.TW93UF* | indole-3-acetic acid inducible 20 |
| AX-177643905 | B09 | *Arahy.R9MXBH* | MYB transcription factor |
| AX-177644205 | B09 | *Arahy.D0SCCK* | MYB transcription factor |
| AX-177644185 | B09 | *Arahy.WDE9JA* | MYB transcription factor |
| AX-177642604 | B09 | *Arahy.Y4YZQQ* | Heat shock protein 21 |
|  | B09 | *Arahy.Z2CVAQ* | Zinc finger (Ran-binding) family protein |

Note:Data comes from Peanutbase,https://dev.peanutbase.org/

Table S5. Analysis of variance for total flavonoid content between C/C and T/T genotypes

| Genotype | df | Mean | SD | SE | F-Value | P-Value |
| --- | --- | --- | --- | --- | --- | --- |
| C/C | 8 | 113.607 | 9.333 | 3.111 | 159.172 | <0.01 |
| T/T | 24 | 74.544 | 7.453 | 1.491 |  |  |

Table S6.Summary of previously reported QTLs for total flavonoid content in peanut

| QTL | Chromosome | Position(cM) | PVE (%) | Marker interval | LOD | Additive |
| --- | --- | --- | --- | --- | --- | --- |
| *qIAA_A02_1* | A02 | 44.6 | 8.10 | mk53 | 3.45 | 576.30 |
| *qIAA_A02_2* | **A02** | **145** | **3.33** | **c2.loc145** | **4.08** | **-2707.00** |
| *qIAA_A03_1* | A03 | 14 | 14.67 | c3.loc14 | 6.78 | -2893.80 |
| *qIAA_A03_2* | **A03** | **88.5** | **44.07** | **mk171** | **21.34** | **3490.50** |
|  | **A03** | **88.8** | **53.75** | **mk170** | **27.40** | **5765.50** |
|  | A03 | 88.8 | 11.16 | **mk170** | 4.19 | -557.40 |
|  | A03 | 89.4 | 27.09 | mk169 | 11.95 | 0.10 |
|  | A03 | 91 | 21.54 | c3.loc91 | 8.30 | -1026.20 |
| *qIAA_A03_3* | **A03** | **152** | **3.64** | **mk135** | **3.95** | **686.10** |
|  | A03 | 153.9 | 15.10 | mk131 | 7.06 | 959.30 |
| *qIAA_A06* | A06 | 15.9 | 9.26 | mk263 | 3.38 | 763.70 |
| *qIAA_A08* | A08 | 0 | 11.79 | mk388 | 4.38 | 860.00 |
| *qIAA_A09* | A09 | 10.9 | 6.13 | mk459 | 2.54 | -582.90 |
| *qIAA_B03_1* | B03 | 21.8 | 2.89 | c13.loc218 | 4.09 | 1636.00 |
| *qIAA_B06_1* | B06 | 24.0 | 7.40 | c16.loc24 | 4.06 | 0.05 |
|  | B06 | 24.0 | 9.08 | c16.loc24 | 3.38 | -5245.00 |
| *qIAA_B06_2* | B06 | 47.2 | 6.68 | mk264 | 2.95 | 2392.70 |
| *qFlv2011-a02* | A02 | 59.3 | 28.0 | S89–TC11D09 | 3.85 | 19.21 |
| *qFlv2010-a03* | A03 | 39.7 | 25.2 | pPGPseq_12A07–UBC841 | 3.15 | -36.2 |
| *qFlv2010-a05* | A05 | 57.4 | 25.0 | GM633–Ah242 | 3.00 | -26.0 |
| *qFlv2010-a05* | A05 | 47.6 | 27.3 | RM14E11–TE389 | 3.42 | 34.4 |
| *qFlv2010-b08* | B08 | 21.0 | 67.0 | ARS796–SSR_HO115593 | 2.90 | -73.26 |

Figure S1. Chromosomal distribution of QTLs and candidate genes associated with total flavonoid content in peanut. QTL distribution map: QTLs associated with total flavonoid content detected in four environments using the RIL population derived from the cross between Silihong and Jinonghei3. E1, E2, E3, and E4 correspond to 2018QY, 2021XS, 2021FX, and 2023XS, respectively. Candidate gene distribution map: Distribution of candidate genes within co-localized intervals (*qflvA06.1*, *qflvA10.1*, *qflvB09.2*) identified from the RIL population (with Silihong and Jinonghei3 as parents) and GWAS across the corresponding chromosomal interval. Genes marked with red boxes indicate candidate genes located at loci mapped in the RIL population for the SNPs identified by GWAS. Genes marked with yellow boxes indicate candidate genes located within 160 kb upstream and downstream of the co-localized intervals between the RIL population and GWAS, including flavonoid-related candidate genes within *qflvA03.1*, which exhibited the highest PVE (16.95%) among QTLs identified in the RIL population.

Figure S2. Line chart of average monthly temperatures in Xushui, Baoding in 2023

Note: https://www.cma.gov.cn/
